# Editorial

**Published:** 1863-12

**Authors:** 


### Editorial.

### CLINICAL CASES IN THE MEDICAL WARDS OF THE MERCY HOSPITAL.

During the past two months, the wards of the Mercy Hospital have been unusually crowded with patients laboring under the most severe and important forms of disease; and the senior class of students in the Chicago Medical College have enjoyed most excellent clinical advantages. During the discussion of continued fevers, in the College, they were enabled to observe in the Hospital, directly at the bedside, the symptoms, progress, and treatment of twelve cases of typhoid fever: illustrating almost every aspect of that disease, with its varied complications.

One of these cases so strikingly illustrated an important practical idea, that we will briefly relate it:—

Mr. —, a native of Ireland; aged about 35 years; laborer; was admitted into the Hospital with typhoid fever, complicated with pneumonic inflammation, involving the middle and lower lobe of the left lung. He had been sick about four days previous to his admission. At that time his face was flushed; lips dry and red; tongue covered with a thick coat, reddish brown and dry in the middle; skin dry and moderately hot; pulse 110 per minute, and soft; abdomen slightly tympanitic, and bowels loose—the discharges being thin and occurring every two or three hours; respiration short and frequent, being somewhat stifled by an acute pain in the infra-axillary region of the left side; some cough and expectoration tinged with blood. Dulness on percussion and sub-mucous rhonchus existed over all the lower half of the left side of the chest. As the typhoid condition of the patient was well marked, and the first stage of the pneumonic inflammation already passed, the treatment directed for the patient was as follows, viz.:—To restrain the typhoid looseness of the bowels, one fluid drachm of the following emulsion was given every four hours:—

℞.—Ol. Terebinth,.................................. ʒij.

Tinct. Opii,.......................................... ʒij.

Pulv. G. Acacia, ........................... } ʒiij.

Sacchar. Alba, ............................ }

Aqua Menthæ,.................................. ℥ij. Mix.

To relieve the pain in the side and counteract the pneumonic inflammation, a blister was applied to the left side of the chest, and one of the following powders given every four hours, alternated with the emulsion:—

℞.—Pulv. Opii, ........................................ 10 grs.
Pulv. Sanguinaria, ............................. 6 grs.
Hydrag. Chlorid. Miti,......................... 6 grs.
Sacchar. Alba, ................................... 30 grs.

Mix and divide into six powders.

Beef tea and milk porridge were directed for nourishment.

At the end of the first 24 hours of the treatment, the calomel was omitted from the powders, and two grains of quinine substituted in its place; but in all other respects the treatment was continued, without change, until the morning of the fourth day. The patient appeared then much improved. The abdomen was not tympanitic; and no movement of the bowels had occurred during the preceding 24 hours. The tongue and skin were moist, and the pulse 90 per minute and soft. There was no pain in the side, except on coughing or taking a forced inspiration, but the left side was still very dull on percussion, with some mucous rhonchus, and expectoration slightly tinged with blood.

The symptoms altogether were so much improved that the previous medicines were discontinued, and a simple anodyne expectorant ordered, as follows:—

℞.—Comp. Honey of Squills, &c.,.................... ℥j.
Tinct. Sanguinaria, .................................... ℥ss.
Tinct. Opii et Camph., .......................... ℥jss.

Mix, and give a teaspoonful every three hours.

Continued animal broth and milk porridge for nourishment. This treatment was ordered on Saturday and continued until Monday, when the clinical class met in the Hospital for the usual hour of instruction at the bedside. On approaching the bed of the patient, it was evident at a glance that he had undergone a decided change since our previous visit. The countenance was expressive of anxiety and restlessness; the face was suffused with a dark purplish redness; the pro-labia were leaden color; the skin on the extremities cool, and capillaries congested; the respiratory movements irregular and feeble; the pulse small, variable, and intermitting; and the impulse of the heart weak. There was constant mental wandering or delirium, with moderate subsultus. The whole left side was dull on percussion. Three or four thin, fecal evacuations had occurred during the preceding 24 hours, with an evident impairment of the action of the sphincter ani. It was evident from the foregoing symptoms, that the patient was rapidly tending towards a fatal degree of depression. By a careful analysis of the symptoms before the class, it was rendered evident that this depression consisted mainly in a failure of the nervous centres, more especially of those of the excito-motory system of MARSHALL HALL, as indicated by the enfeebled respiration and circulation; and in impaired contractility of the muscular tissues, with imperfect decarbonization of the blood. The enfeebled action of the heart, accompanying such a condition as here described, and occurring during the progress of typhoid fever, is highly dangerous, and not unfrequently productive of unexpectedly fatal results. To counteract this condition, the liberal use of alcoholic stimulants is very generally resorted to by the profession. And if the lungs are free from obstruction, they will sometimes produce the desired effect. But their constant tendency to retard atomic changes in the tissues, and lessen the elimination of carbonic acid gas from the lungs, renders them inapplicable and often positively injurious in such cases as the one under consideration, in which pneumonic obstruction exists throughout the whole of one lung and the blood very imperfectly decarbonized. Does the materia medica afford us any remedy which, by its efficient action on the involuntary nervous centres and on the contractility of the muscular and fibrous tissues, is specially adapted to the present case? The therapeutic properties of strychnine are such as to render it more perfectly applicable to the treatment of such a case than any other remedy with which we are acquainted. For several years, we have been in the habit of resorting to it in all cases of typhoid disease, during the progress of which there occurred unusual depression of the excito-motory nervous functions, as indicated by feebleness of respiration, feebleness of the heart's action, and impairment of the sphincters; and generally with prompt benefit. In the present case, we directed the emulsion to be continued in doses of one fluid drachm every four hours, with two grains of tannate of quinine in each dose; and the strychnine to be given alternately with it, as follows:—

℞.—Strychnine, .................................. 1 gr.

Nit. Acid, .................................. ʒj.

Water, .................................. ℥ij.

Mix, and give one teaspoonful in a little sweetened water every four hours. Continue beef-tea and milk porridge for nourishment.

In twenty-four hours the heart's action had become steady and uniform, although the pulse was still small and weak. The expression of countenance and the respiratory movements were also improved; but the delirium and sleeplessness continued, with much dulness in the left side, and expectoration tinged with blood. The bowels continued quiet. The strychnine and nitric acid solution was continued, but, instead of the emulsion and quinine, he was directed a powder of pulv. Doveri, 8 grs., and pulv. g. camph., 2 grs., between the doses of strychnine, and an additional blister to the side. From this time the delirium steadily diminished; the patient had intervals of quiet sleep; the respiration and circulation improved daily; and in one week from the time he commenced the strychnine he was convalescent; although there remained some dulness over the left side, with moderate cough and expectoration. These disappeared during the succeeding week, under the use of mild anodyne expectorants, and the patient was discharged.

CASE II.—Mr. C., aged about 25 years, was admitted into ward No. 7, Mercy Hospital, Nov. 21st, complaining of severe aching pains in his head, back, and limbs, with a harsh bronchial cough, accompanied by a sense of soreness and constriction in the central part of the chest. The tongue was partially covered with a white fur; the skin dry and above the natural temperature; pulse 95 per minute, moderately firm; respirations slightly increased in frequency; and bowels quiet. On close inquiry it was found that distinct rheumatic inflammation and swelling existed in the ankles and knees, and in one wrist. But neither auscultation nor percussion afforded any evidence of the existence of pneumonic inflammation. Regarding the case as one of sub-acute rheumatism complicated with bronchitis, we directed a powder composed of eight grains each, of pulv. Doveri and nit. potassa, with two grains of calomel, every four hours; and a mild anodyne expectorant between. On the 22d, the symptoms were not materially changed, and the same treatment was continued, with the addition of a saline cathartic. On the 23d, the clinical class visiting that ward, examined his case minutely; and their attention was called to the fact that it represented a class of cases at that time occurring in the city with unusual frequency. And in some of which the inflammation, after four or five days, seemed to extend suddenly from the bronchial ramifications to the lobules of the lungs, causing great dyspnœa; a small and very frequent pulse; leaden hue of the lips; great feeling of weakness; and in a few hours, extensive dulness on percussion, with coarse mucous rhonchus.

In a few of these cases, the air cells have become so rapidiy and universally compressed, that the patients have died, with symptoms of apnœa, within 24 hours after the first indications of pneumonic congestion. In another class of cases, the patients, instead of complaining of rheumatic pains and swellings in the articulations, have exhibited the symptoms of an attack of simple catarrh, with some cough and sense of tightness in the chest for three or four days, but not sufficiently severe to prevent them from being up and attending moderately to their ordinary duties. Then a very acute pain would attack the sub-axillary region of one side, greatly aggravated by a full inspiration or a cough, and accompanied by a moderate grade of general fever. At first, neither auscultation nor percussion indicated any decided change in the lungs. But within 24 hours, a crepitant or sub-crepitant rale became plainly audible over the whole lower lobe of the lung, with moderate dulness on percussion, and bloody expectoration. If not promptly checked by treatment, in 24 hours more, the whole of the affected side would become dull on percussion; a sub-mucous rhonchus equally extensive, with expectoration more largely mixed with blood; the breathing laborious; the pulse from 120 to 130 per minute, soft and weak; the lips leaden color; the skin moist; and extremities cool; with dulness of mental faculties, and great sense of exhaustion. In a very few cases, both lungs became involved almost simultaneously, and produced a fatal result in less than 48 hours. In a large majority of the cases, however, the disease remains limited to one lung, and the patients slowly recover.

The class were informed that although there were no alarming symptoms then present in the case before them, yet, from the extent of the bronchial inflammation, and the known tendency of similar cases to extend suddenly to the lobules of the lungs, a very cautious prognosis must be given.

Believing the pulmonary inflammation, like that in the articulations, to be rheumatic in its nature, the patient was directed the following treatment:—

℞.—Vin. Colchici Sem., .......................... ℥j.

Acetum Opii,.......................................... ℥ss.

Mix, and give 30 drops every four hours, alternately with a powder containing pulv. Doveri eight grains, nit. potassa eight grains, and calomel one grain.

The next day, the 24th, the cough was less severe, and the pains in the limbs less, but otherwise the symptoms remained the same. The same treatment was continued.

On making the visit at the usual time, 1 o'clock P.M., on the 25th, the aspect of the patient was entirely changed. A complete apoplectic engorgement of both lungs had ensued; causing great difficulty and shortness of breathing; a cool skin; livid face and lips; pulse weak, irregular, and small; mental faculties dull; and a coarse mucous ronchus, with dulness on percussion all over the chest.

No further treatment had any effect upon him; he died before the next morning.

---

The following interesting letter was received too late to go into the original department of the present number.—[Ed.]

### ICE AS A THERAPEUTIC AGENT IN AFFECTIONS OF THE THROAT.

---

By M. K. TAYLOR, Surgeon U. S. Vols., and Professor of Theory and Practice of Medicine in the Medical Department of the Iowa State University.

---

Prof. N. S. Davis,

Dear Sir,—I have noticed several paragraphs in the public journals lately, referring to the employment of ice, by some French gentleman, whose name I do not at this moment recollect, in certain affections of the throat. His mode of applying the ice seems to be that of allowing it to be dissolved slowly in the mouth, or of swallowing it that it might be dissolved in the stomach. I have no doubt of its efficacy in many cases when thus used. There are many instances, however, and particularly in infants, when it is difficult to secure any such favorable results, because of the want of coöperation on the part of the patients. A more practicable mode, and one with which I have been very favorably impressed, after some four or five years' trial, is that of its external application to the throat, in nearly all of the local inflammations of that region, not connected with the eruptive fevers.

I have used in both inflammatory and spasmodic croup, in diphtheria, tonsilitis laryngitis, œdema of the glottis, and I assure you of my belief that we possess no remedy so effective, and at the same time so manageable, as the external application of ice to the larynx or parts higher up, when thus inflamed. Its powerful sedative impression is observed in a very short time directly upon the morbid process, while there is a general sedation seen in the diminished action of the heart and loss of temperature, with a corresponding modification of febrile excitement upon the continuance of the application of the remedy. In infants, I have seen it control the croupy respiration in a very few minutes, and that too when time is of the utmost importance, as in the severe forms of the spasmodic variety. In diphtheria, it does not always arrest the exudation of false membrane, but the ice will diminish the amount thrown out, and assuage the local pain and swelling very much. In the earlier stage of tonsilitis it will often arrest the disease, always modifies and lessens the inflammatory action, and prevents, to a very considerable extent, the suppurative process. In some cases, however, when repeated suppurative inflammations have occurred in the tonsils before, it has not always arrested the formation of an abscess—perhaps it might have done so if applied in an earlier stage of the disease. My mode of application has been to secure a piece of ice, the size of a hen's egg, so shapen as to adapt itself to the form of the neck, upon each side of the larynx, or as near the seat of inflammation as practicable; and for tonsilitis immediately to the sub-maxillary region, upon one or both sides, as the case might require.

I have generally adjusted the ice by enveloping it in a single thickness of oiled silk so that it could not slip from its proper place, by adjusting it saddlewise over the larynx, and then envelope the whole neck with several thicknesses of flannel, with the view of preventing the temperature of the surrounding air from contributing to any extent in dissolving it. When the ice seems to be no longer required, the moderate application of cold water will prevent too great reaction, and the lighting up anew of the morbid action.

It does not, or at least I have not relied upon it solely with that view, do away the necessity of other treatment; but I have generally employed such medication as the circumstances seemed to demand for the arrest of the disease, with only this precaution: that antimony and viratrum be administered sparingly, lest too great depression be obtained.

It will be recollected that the ice lies closely upon the larger vessels of the neck, and that the greater part of all the blood sent to and returning from the brain comes more or less under its influence; and that the sedative effect of the small quantities thus employed is much more marked than when a considerable larger quantity is applied to the whole cerebrum.

I have not time to prepare notes of cases, if I were so disposed, because of the pressure of my public duties; nor do I consider it particularly necessary to ensure the trial of the remedy by the profession at large. The known sedative action of cold is too well appreciated by the profession to require such demonstration.

I have not employed it in those anginose affections of the throat connected with scarlatina, lest it might interfere with the appearance of the eruption; though in a desperate case, when other remedies had failed, I should do so, and seek to counteract any unpleasant effect by friction to the surface, and artificial heat to the remote parts. I have seen no unpleasant effects from its use, though I can readily conceive that on young infants, without proper care, its action might be carried too far.

---

### GLEANINGS FROM THE FRENCH JOURNALS.

By the Editor.

*Influence of Steam on Lead.*—M. Lermer, in Dingler's *Polytech. Journ.*, has published an interesting article on the corroding influence of aqueous vapor on lead pipes. This effect is found to greatly increase in proportion to the purity of the lead. By alloying the lead with tin, the action of the vapor is much reduced, and at a minimum when the lead is but 37 per cent of the mass.—*Jour. de Chim. Med.*

*Arsenic in Sulphuret of Antimony.*—An ounce of the black sulphuret of antimony was given to twenty-four sheep, of which number ten died. On examining the sulphuret by Wackenroder's process, M. Reynolds discovered 1.33 per cent of sulphuret of arsenic.—*Jour. de Chim. Med.*

*Iodide of Iron and Quinia, crystalized.*—M. Smedt believes that he has obtained this salt perfectly definite. He prepares it as follows: Take of sulphuret of barium a sufficient quantity. Make a concentrated solution in water, precipitate it by tincture of iodine, filter to separate the sulphur, and add 30 parts of sulphate of quinia dissolved in concentrated alcohol.

Sulphate of baryta precipitates and iodide of quinia remains dissolved in the alcohol and communicates to it a deep yellow color; thrown on a filter, the sulphate of baryta is washed with alcohol, the liquors, united and evaporated, yield the iodide of a beautiful orange yellow color; lastly, 12 parts of iodine are transformed into a concentrated solution of iodide of iron, to which the alcoholic solution of iodide of quinia is added, and heated on a water bath. As the alcohol evaporates, the liquor assumes a beautiful green color, and a small quantity of dark green resinous matter separates. Towards the end of the evaporation a little alcohol is again added, filtered, and left to crystalize; the crystals are expressed strongly and dried.

The iodide of iron and quinia obtained by this means is in long needles of a beautiful yellow color, completely soluble in boiling water, and not precipitating on cooling. This salt dissolves in cold alcohol and ether, is without odor, and has a bitter and ferruginous taste. In fact, it presents all the characters of a perfectly definite compound, but its composition has not been verified by analysis.—*Jour. de Chim. Med.*, Sept. 1863.

*Falsification of Essence of Mace.*—M. de Letter has noticed a fraud which consists in substituting an alcoholic tincture of nutmegs for the essential oil. This product has a golden yellow color, and is very fluid, two properties which are not characteristic of the essential oil of mace. Besides, this pretended essence mixes with water, rendering it slightly lactescent, like tincture of nutmegs, which in color resembles the fraudulent essence. A few drops of the latter tested with bichromate of potassa and sulphuric acid developes instantly the green coloration due to alcohol.—*Bul. Soc. de Pharm. de Brux.* et *Jour. de Chim. Med.*

*Syrup of Balsam Copaiba.*—M. Ed. DuMay (*Jour. de Chim. Med. Aout*, 1863) gives the following receipt for this syrup:—

| | | |
|---|---|---|
| Take of Balsam of Copaiba, of Cayenne,... | 167 | grammes. |
| Calcined Magnesia,..................... | 9 | " |
| Simple Syrup,.......................... | 320 | " |
| Yolk of Egg, fresh,.................... | 4 | |

Triturate the yolk of eggs with the magnesia, and add afterwards and mix intimately the copaiba, and finally the syrup. This preparation keeps well.

*Casein Cement.*—Dr. Wagner recommends the employment of a cold saturated solution of borax or of silicate of soda, to dissolve casein in preference to the alkaline carbonate indicated by Braconnot. The solution of casein by borax is a clear liquid, of viscid consistence, more adhesive than gum, and able to replace in many cases strong glue. Stuffs of linen and cotton impregnated with this solution can be treated with tannic acid or acetate of alumina and rendered impermeable. Marsden, in his *History of Sumatra*, has shown that the chief cement employed in that country is made from the curdled buffalo's milk, and called *prackee.* To prepare it, the milk is abandoned to itself until the cream becomes butter, which is removed by a spoon and washed with water for use. The residual liquid of the milk is sour and thick, and it is this that they call *prackee.* They press it strongly so as to get it into the form of cakes, which are dried and become excessively hard. When it is to be used, a certain quantity is scraped off, mixed with quick lime in powder and moistened with milk. The cement thus obtained is extremely solid, and resists perfectly hot and humid climates a great deal better than glue; it is specially good for cementing porcelain.—*The Technologist. Jour. de Chim. Med.*

The following recipes for mineral salts have been translated from the Report of MM. Bussy, Grandeau and Baudrimont, to the Commission revising the French Codex:—

*Sulphate of Protoxide of Manganese.*

| | | |
|---|---|---|
| Take of Black Oxide of Manganese (natural),...... | 1 | part. |
| Commercial Proto-sulphate of Iron,........ | 1 | " |

By contusion and trituration make an intimate mixture and heat in an earthen crucible to ordinary redness. The mass being cooled, pulverize it and exhaust it with boiling water and evaporate to dryness. Redissolve the residue in hot water, filter and evaporate and crystalize.

This salt is very soluble in water, and should not be colored blue by yellow prussiate, nor black by sulphuretted hydrogen, after adding acetate of soda.

*Chlorate of Soda.*

| | | |
|---|---|---|
| Take of Crystalized Tartaric Acid,............... | 150 | parts. |
| Carbonate of Soda, crystalized,......... | 143 | " |
| Chlorate of Potassa,........................ | 121 | " |

Dissolve the tartaric acid and carbonate of soda separately, in convenient quantities of hot water, and add little by little the solution of carbonate, to that of the acid in a capsule sufficiently large to prevent the liquid from loss by effervescence. When this last has ceased, agitation causes the deposition of the bitartrate of soda.

On the other hand, dissolve the chlorate of potassa, in twice its weight of boiling water, and mix this solution with the magma of the bitartrate of soda resting in the capsule. Carry the whole to ebullition, and add enough of water to dissolve and allow it to cool completely. Filter the liquor to separate the precipitate of cream of tartar which forms. Evaporate the filtrate, nearly to dryness, and stir it to avoid loss by decomposition. When cold lixiviate the granular salt with four or five times its weight of cold water, agitating to facilitate solution, and when there only remains a granular crystalline residue which refuses to dissolve, filter and evaporate to one-fourth, and set aside to crystalize.

Thus made, this salt ought not to blacken when heated on platina foil, and should but slightly trouble a solution of nitrate of silver. It is soluble in three times its weight of cold water. 122 parts of chlorate of potassa yield 106 parts of chlorate of soda.

*Hypermanganate of Potassa.*—KO, $Mn^2 O^7$.

Take of Binoxide of Manganese, finely pulverized and washed,........................................ 10 parts.
Caustic Potassa, well fused,................ 12 "

Put them in a cast iron basin, add a little water, sufficient to render the mixture pasty, dry rapidly, stirring constantly with a strong iron spatula whilst heating over the fire, to remove all humidity; introduce the grumous mass obtained, into a tubulated earthen retort, and lute in its tubulure a large green glass tube, descending nearly to the bottom of the retort. Place the retort in the upper part of a laboratory furnace, adapt to its neck a curved tube, which shonld dip two centimetres into mercury. Pass through the tube, dipping into the retort, a current of oxygen deprived of carbonic acid gas, at the moment when the retort has attained a dull, red heat. The action is terminated when the oxygen is disengaged freely through the tube dipping in the mercury, or when it ceases to disengage watery vapor.

The retort is now cooled, emptied, and the contents lixiviated with a sufficient quantity of warm water. Pass into the liquor thus obtained, a current of washed carbonic acid, until the solution has taken the characteristic violet tint of the hypermanganate. Allow it to repose 24 hours, decant and rapidly evaporate it without ebullition to the proper degree, that, by cooling, it will make a good crystallization of hypermanganate. The mother waters are evaporated whilst they yield prismatic crystals by evaporation. They are carefully dried, avoiding the contact of organic matter.

100 parts of binoxide, yield 35 to 40 parts of permanganate in the first crystallization.

This salt is in beautiful prismatic needles, of a bronzed and violet black. It should be entirely soluble in water. Its solution of a magnificent violet becomes green by alkalies.

---

### ROYAL HUMANE SOCIETY'S INSTRUCTION'S.

### DIRECTIONS FOR RESTORING THE APPARENTLY DEAD:

I.—*If from Drowning or other Suffocation, or Narcotic Poisoning.*—Send immediately for medical assistance, blankets, and dry clothing; but proceed to treat the patient *instantly*, securing as much fresh air as possible.

The points to be aimed at are, first and immediately, the restoration of breathing; and second, after breathing is restored, the promotion of warmth and circulation.

The efforts to restore life must be persevered in until the arrival of medical assistance, or until the pulse and breathing have ceased for at least an hour.

### TREATMENT TO RESTORE NATURAL BREATHING.

RULE 1.—*To maintain a free entrance of Air into the Windpipe.*—Cleanse the mouth and nostrils; open the mouth; draw forward the patient's tongue, and keep it forward: an elastic band over the tongue and under the chin will answer this purpose. Remove all tight clothing from about the neck and chest.

RULE 2.—*To adjust the Patient's position.*—Place the patient on his back on a flat surface, inclined a little from the feet upwards; raise and support the head and shoulders on a small firm cushion, or folded article of dress, placed under the shoulder-blades.

RULE 3.—*To imitate the movements of Breathing.*—Grasp the patient's arm just above the elbows, and draw the arms gently and steadily upwards, until they meet above the head, (this is for the purpose of drawing air into the lungs), and keep the arms in that position for two seconds. Then turn down the patient's arms, and press them gently and firmly for two seconds against the sides of the chest, (this is with the object of pressing air out of the lungs. Pressure on the breast-bone will aid this.)

Repeat these measures alternately, deliberately, and perseveringly, fifteen times in a minute, until a spontaneous effort to respire is perceived, immediately upon which cease to imitate the movements of breathing, and proceed to induce circulation and warmth, (*as below.*)

Should a warm bath be procurable, the body may be placed in it up to the neck, continuing to imitate the movements of breathing. Raise the body in twenty seconds in a sitting position, and dash cold water against the chest and face, and pass ammonia under the nose. The patient should not be kept in the warm bath longer than five or six minutes.

RULE 4.—*To excite Inspiration.*—During the employment of the above method, excite the nostrils with snuff or smelling salts, or tickle the throat with a feather. Rub the chest and face briskly, and dash cold and hot water alternately on them.

*** The above directions are chiefly Dr. H. R. Silvester's method of restoring the apparently dead or drowned, and have been approved by the Royal Medical and Chirurgical Society.

TREATMENT AFTER NATURAL BREATHING HAS BEEN RESTORED.

RULE 5.—*To induce Circulation and Warmth.*—Wrap the patient in dry blankets, and commence rubbing the limbs upwards, firmly and energetically. The friction must be continued under the blankets or over the dry clothing.

Promote the warmth of the body by the application of hot flannels, bottles or bladders of hot water, heated bricks, &c., to the pit of the stomach, the armpits, between the thighs, and to the soles of the feet. Warm clothing may generally be obtained from the bystanders.

On the restoration of life, when the power of swallowing has returned, a teaspoonful of warm water, small quantities of wine, warm brandy-and-water, or coffee, should be given. The patient should be kept in bed, and a disposition to sleep encouraged. During reaction, large mustard plasters to the chest and below the shoulders will greatly relieve the distressed breathing.

II.—*If from Intense Cold.*—Rub the body with snow, ice, or cold water. Restore warmth by slow degrees. In these accidents it is highly dangerous to apply heat too early.

III.—*If from Intoxication.*—Lay the individual on his side on a bed, with his head raised. The patient should be induced to vomit. Stimulants should be avoided.

IV.—*If from Apoplexy or Sun-stroke.*—Cold should be applied to the head, which should be kept well raised. Tight clothing should be removed from the neck and chest.

APPEARANCES WHICH GENERALLY INDICATE DEATH.

There is no breathing or heart's action; the eyelids are generally half-closed; the pupils dilated; the jaws clenched; the fingers semi-contracted; the tongue appearing between the teeth; and the mouth and nostrils are covered with a frothy mucus. Coldness and pallor of surface increase.

---

SCLEROTITIS AND IRITIS.—The plan of internal administration of morphia in acute cases of sclerotitis, continues to be as successfully pursued as heretofore. At the commencement of a case, doses of a-quarter of a grain should be given, cautiously increased to one-third or even half a grain, but in some young or feeble subjects it is well to begin with one-fifth of a grain. The patient should be directed to take one dose every third hour; but on the pain becoming less, to increase the interval of the doses to four or five hours, or even to leave off the medicine altogether. The degree of pain should be the guide as to the length of interval. A mercural purge and tonics may be required at the termination of the case. In a few cases, morphia causes violent symptoms of stomach derangement and depression; in others it produces these effects but in a slight degree; in such cases the drug is inadmissable, except in very small doses. Where there is iritis, belladonna should be applied locally. The important practical fact seems established, that morphia is, *per se*, a powerful antiphlogistic, capable of curing those acute inflammations of the eye in which, up to the present time, blood-letting, blistering, and mercurialisation have been considered necessary. The explanation of this remarkable action of morphia in reducing abnormal fulness of the vessels of the sclerotic, we may find in the relations of pain to vascular congestion. It is generally considered that the pain is the effect of congestion, but it is quite an open question whether, in certain classes of cases, the order of things may not be reversed. (Dr. J. C. Lawrence, page 175.)

---

INVESTIGATIONS TOUCHING THE USE OF IODINE.—Dr. Rosenthal, assistant physician at the Vienna General Hospital, has published, in the *Wiener Med. Wochensch.*, a series of papers containing much original matter touching the therapeutic use of iodine. The summing up is as follows:—

1. Large doses of iodide of potassium, combined with a small quantity of fluid, remain a long time in the economy; with large quantity of fluid, they are quickly washed away from the system, and pass rapidly into the secretions and excretions. This circumstance should be carefully noticed.

2. When iodide of potassium is taken internally, it is found not only in the urine, saliva, and other secretions, but also in the alvine evacuations, within from four to seven hours, whether the stools be aqueous or the reverse.

3. In the administration of iodide of iron, iodine is separated in considerable quantities, and found with a large proportion of the iron in the urine. Fæcal matter contains much iron and a small amount of iodine. The same phenomena may be noticed when iodide of mercury is used.

4. Frictions with an ointment containing iodide of potassium upon sound skin will cause the iodine to be detached in the urine and saliva.

5. Iodine is found in the urine of those who take baths in which iodide of potassium is dissolved, even when the rectum and urethra are kept free from the action of the bath. This is proved by examining the urine, and by noting a large diminution of the iodine in the water used for the bath.

6. The intestinal mucous membrane takes of the iodine very energetically in the form of enemata, and this is the case even with very weak solutions of iodide of potassium.

7. Large doses of iodide of potassium, or small doses taken for a long time, are not well borne in certain pathological states of the economy; in fact, large doses of iodine, or concentrated solutions, are very prejudicial to the system.

---

Inquests of 1862.—The coroners' returns show that 20,591 inquests were held in England in the year 1862—a number slightly below the average—14,198 on males, and 6393 on females. There were 221 verdicts of murder, 207 of manslaughter, 1284 of suicide, 2429 of "found dead," 157 of death from want, cold, and exposure. The number of inquests held on children under seven years of age was 6002—1107 of the number on illegitimate children; and there were 3239 inquests on infants not more than a year old, of whom 839 were illegitimate. This is the first year in which the children of illegitimate birth have been distinguished in these returns. More than a-sixth of the children on whom inquests were held were illegitimate, more than a-fourth of the infants not above a year old. The verdicts of wilful murder numbered 124, more than half of which related to children not more than the age of twelve months.

HEALTH OF SAN FRANCISCO.—*Comparative Mortality for the Two Years ending respectively August* 31, 1862 *and* 1863.

The total number of deaths for the month of August was 163, being 17 less than for the preceding month. 95 were recorded in the Lone Mountain cemetery, of which 23 were under three years; 66 in the Roman Catholic burying ground, of which 23 were under three years; and 2 in the Jewish cemeteries. The principal causes of death are recorded as follows: Consumption; 12; lung diseases, 3; brain diseases, 6; apoplexy, 2; heart diseases, 3; fever, 2; typhus fever, 2; enteritis, 1; dropsy, 2; cholera infantum, 2; sore throat and diphtheria, 6; croup, 1; scarlatina, 2; dentition, 2; whooping cough, 3; measles, 2; erysipelas, 1; debility, 4; paralysis, 3; still born, 15.

*The following is a comparative table of mortality for the two years ending respectively August* 31, 1862 *and* 1863:—

| 1861. | | 1862. | |
|---|---|---|---|
| September, | 164 | September, | 170 |
| October, | 153 | October, | 173 |
| November, | 165 | November, | 167 |
| December, | 195 | December, | 179 |
| 1862. | | 1863. | |
| January, | 173 | January, | 162 |
| February, | 218 | February, | 151 |
| March, | 207 | March, | 192 |
| April, | 174 | April, | 157 |
| May, | 152 | May, | 173 |
| June, | 160 | June, | 175 |
| July, | 199 | July, | 180 |
| August, | 222 | August, | 163 |
| Total, | 2183 | Total, | 2040 |

REMARKS.—Notwithstanding the steady increase of our population, it will be perceived that there was 143 deaths less, for the year ending August 31, 1863, than in the preceding year.

The average monthly mortality for the year ending August, 1862, was 182; for the last year it was only 170. Of the 2040 who died during the last twelve months, 551 were children under 3 years of age, and 140 still born.

During the last year, the highest monthly mortality was in March—192; the lowest in February—151. During the previous year, the highest monthly mortality was in August—222; the lowest in May—152.

If we estimate our present population at 100,000 our rate of mortality has been less than 1 in 49; showing a degree of salubrity which may be advantageously compared with that of any large city in the civilized world.—*Pacific Med. & Surg. Jour.*

